# The Extracellular Domains of GluN Subunits Play an Essential Role in Processing NMDA Receptors in the ER

**DOI:** 10.3389/fnins.2021.603715

**Published:** 2021-03-16

**Authors:** Martin Horak, Petra Barackova, Emily Langore, Jakub Netolicky, Paula Rivas-Ramirez, Kristyna Rehakova

**Affiliations:** Department of Neurochemistry, Institute of Experimental Medicine of the Czech Academy of Sciences, Prague, Czechia

**Keywords:** disulfide bridges, glutamate receptor, glycosylation, excitatory synapse, posttranslational modification

## Abstract

*N*-methyl-D-aspartate receptors (NMDARs) belong to a family of ionotropic glutamate receptors that play essential roles in excitatory neurotransmission and synaptic plasticity in the mammalian central nervous system (CNS). Functional NMDARs consist of heterotetramers comprised of GluN1, GluN2A-D, and/or GluN3A-B subunits, each of which contains four membrane domains (M1 through M4), an intracellular C-terminal domain, a large extracellular N-terminal domain composed of the amino-terminal domain and the S1 segment of the ligand-binding domain (LBD), and an extracellular loop between M3 and M4, which contains the S2 segment of the LBD. Both the number and type of NMDARs expressed at the cell surface are regulated at several levels, including their translation and posttranslational maturation in the endoplasmic reticulum (ER), intracellular trafficking *via* the Golgi apparatus, lateral diffusion in the plasma membrane, and internalization and degradation. This review focuses on the roles played by the extracellular regions of GluN subunits in ER processing. Specifically, we discuss the presence of ER retention signals, the integrity of the LBD, and critical *N*-glycosylated sites and disulfide bridges within the NMDAR subunits, each of these steps must pass quality control in the ER in order to ensure that only correctly assembled NMDARs are released from the ER for subsequent processing and trafficking to the surface. Finally, we discuss the effect of pathogenic missense mutations within the extracellular domains of GluN subunits with respect to ER processing of NMDARs.

## Introduction

**FIGURE 1:**
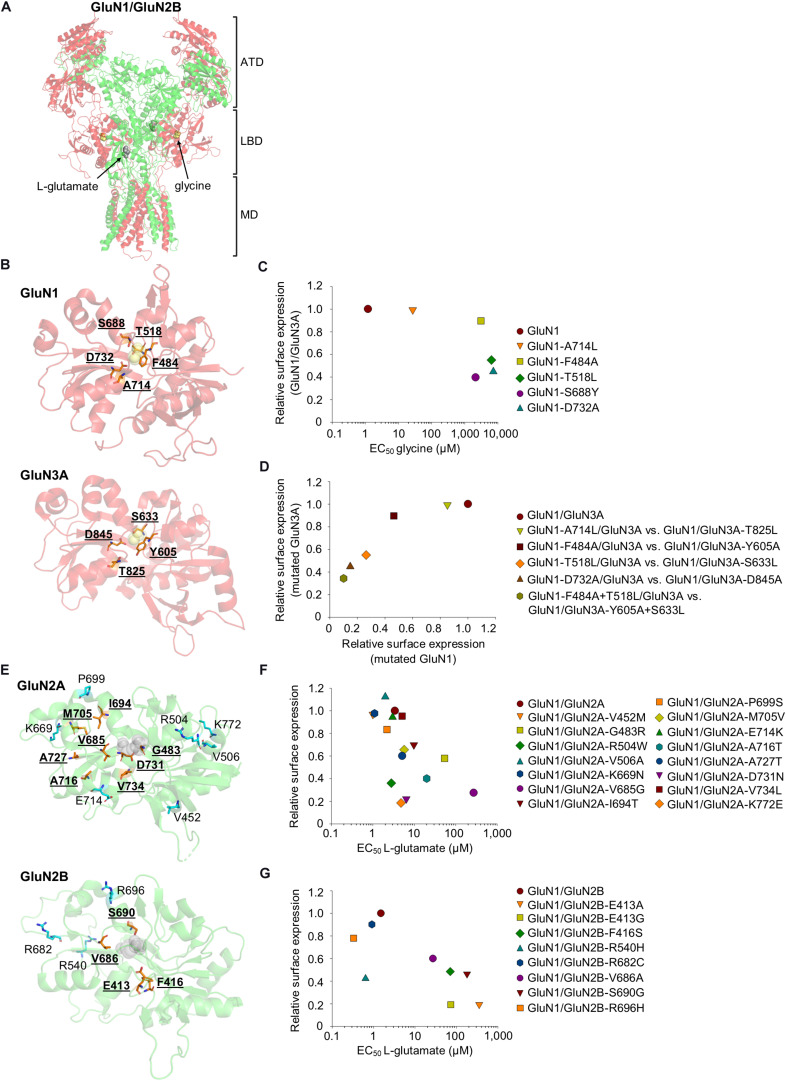
**(A)** The crystal structure of the GluN1/GluN2B heterotetramer (PDB code: 5IOU) (Zhu et al., 2016), including the glycine (yellow) and L-glutamate (gray) molecules. ATD, amino-terminal domain; LBD, ligand-binding domain; MD, membrane domain (the C-terminal domain is not shown). The GluN1 is shown in red, and the GluN2B is shown in green. **(B,E)** Schematic depiction of the LBD in the GluN1 (PDB code: 1PB7), GluN3A (PDB code: 2RC7), GluN2A (PDB code: 5H8Q), and GluN2B (PDB code: 5IOU), including the glycine [yellow, **(B)**] and L-glutamate [gray, **(E)**] molecules. The amino acid residues reviewed in the text are indicated, and residues included in the published series of mutant NMDARs with altered EC_50_ values for glycine/L-glutamate and surface expression are shown in orange. **(C)** The relationship of surface expression of mutated GluN1 co-expressed with wild-type GluN3A (Skrenkova et al., 2019), the EC_50_ values for glycine were obtained using GluN1/GluN2A receptors (Williams et al., 1996; Kvist et al., 2013; Skrenkova et al., 2020). **(D)** The relationship of surface expression of GluN1/GluN3A receptors with analogous mutations in GluN1 and GluN3A (Skrenkova et al., 2019). **(F,G)** The relationship of surface expression of the indicated GluN1/GluN2A **(F)** or GluN1/GluN2B **(G)** receptors with their EC_50_ values for L-glutamate (Laube et al., 1997; She et al., 2012; Swanger et al., 2016). If necessary, the values of the relative surface expression of NMDARs were obtained by calculating values from graphs from the publications using ImageJ 1.52N software (National Institutes of Health, Bethesda, Maryland, United States) (Schneider et al., 2012).

*N*-methyl-D-aspartate receptors (NMDARs) are ionotropic glutamate receptors that play an essential role in mediating excitatory neurotransmission (Traynelis et al., 2010; Vieira et al., 2020). NMDARs are heterotetramers comprised of two GluN1 (with eight splice variants) and two GluN2 (GluN2A through GluN2D) and/or GluN3 (GluN3A and GluN3B) subunits (Paoletti, 2011; Perez-Otano et al., 2016). All GluN subunits contain four membrane domains (M1 through M4), an extracellular amino-terminal domain (ATD) and the S1 segment of the ligand-binding domain (LBD), an extracellular loop between M3 and M4 containing the S2 segment of the LBD, and an intracellular C-terminal domain (CTD) (Traynelis et al., 2010; Paoletti et al., 2013; Figure 1A). It has been well established that the S1 and S2 segments grasp their specific amino acid ligands in the cleft and close around it after its binding, resembling a Venus fly trap or a clamshell-like domain (Felder et al., 1999; Traynelis et al., 2010). The conventional NMDAR subtype—comprised of GluN1 and GluN2—is activated upon binding an agonist such as L-glutamate to the LBD of GluN2 (hereafter called the “glutamate-binding site”) together with a co-agonist such as glycine to the LBD of GluN1 (hereafter called the “glycine-binding site”). The unconventional NMDAR subtype—comprised of GluN1 and GluN3—is activated by agonist binding to the glycine-binding site of GluN3, with desensitization mediated by binding of a co-agonist to the glycine-binding site of GluN1. There were also found the triheteromeric NMDARs such as GluN1/GluN2A/GluN2B and GluN1/GluN2/GluN3A receptors with functional and pharmacological properties different from diheteromeric NMDARs (Perez-Otano et al., 2016; Stroebel et al., 2018). Thus, the NMDARs are composed of various combinations of GluN subunits, which dictates their functional properties.

Surface numbers and types of NMDARs are dynamically regulated by the balance between their exocytosis and internalization (Wenthold et al., 2003; Vandenberghe and Bredt, 2004; Horak et al., 2014). The biogenesis of NMDAR begins with the transcription of the *GRIN* subunit genes, followed by their translation in the rough ER. It has been shown that a large amount of unassembled GluN1 is present in the ER in mammalian neurons, whereas both GluN2A and GluN2B are expressed in limited numbers (Chazot and Stephenson, 1997; Huh and Wenthold, 1999). Unassembled GluN1-1, GluN2, and GluN3 are retained in the ER due to the presence of ER retention signals in the CTD of GluN subunits (Okabe et al., 1999; Perez-Otano et al., 2001), such as the KKK and RRR motifs in the C1 cassette of the GluN1 (Standley et al., 2000; Scott et al., 2001; Horak and Wenthold, 2009), HLFY motif in the proximal part of the CTD of the GluN2B (Hawkins et al., 2004) as well as the RXR motif in the GluN3B (Matsuda et al., 2003). In addition, ER retention signals in the membrane domains of the GluN1, GluN2A and GluN2B (Horak et al., 2008), as well as in the NTDs of the GluN subunits (see below) are also likely used. Interestingly, the presence of the PSD-95, Dlg, and Zo-1 (PDZ)-binding motif at the distal end of the CTD of the GluN1-3 splice variant negates both ER retention signals in the C1 cassette, likely due to its interaction with postsynaptic density (*PSD*)-95 family of membrane-associated guanylate kinases (PSD-MAGUKs) and/or coat protein complex II (COPII) (Standley et al., 2000; Scott et al., 2001; Mu et al., 2003). Given the fact that splicing of GluN1 is regulated by synaptic activity (Mu et al., 2003), it is clear that the processing of unassembled GluN subunits in the ER is a complex and highly regulated process. Several models have been proposed to describe the assembly of functional NMDAR heterotetramers in the ER, including the involvement of GluN1-GluN1 and GluN2-GluN2 homodimers (Meddows et al., 2001; Schorge and Colquhoun, 2003; Papadakis et al., 2004; Qiu et al., 2005) or GluN1-GluN2 heterodimers (Schuler et al., 2008). On the contrary, another model predicts that GluN1-GluN1 homodimers are essential for oligomeric assembly with the GluN2 subunit (Atlason et al., 2007). The assembled NMDAR heterotetramers are further processed by ER quality control machinery, which likely controls that they are in the correct conformation, as well as that all ER retention signals within the GluN subunits are properly negated. NMDARs then likely bypass the somatic Golgi apparatus (GA), and are processed in dendritic Golgi outposts (Jeyifous et al., 2009), from which they are delivered in vesicles to extrasynaptic membranes *via* exocytosis (Gu and Huganir, 2016). Surface NMDARs can be anchored by postsynaptic density through lateral diffusion (Tovar and Westbrook, 2002; Groc et al., 2004, 2006) or undergo endocytosis, recycling and degradation (Roche et al., 2001; Nong et al., 2003; Lavezzari et al., 2004; Scott et al., 2004). There are many comprehensive reviews that cover events that occur during NMDAR transport to and from cell surface membranes, especially focusing on the CTD domains of GluN subunits (Wenthold et al., 2003; Vandenberghe and Bredt, 2004; Lau and Zukin, 2007; Petralia et al., 2009; Horak et al., 2014; Perez-Otano et al., 2016; Vieira et al., 2020). The focus of this review is to summarize and discuss the most up-to-date knowledge regarding the ER processing of NMDARs, with an emphasis on the role of the extracellular GluN domains. A major hypothesis in this field is that the ER quality control machinery senses the proper ligand occupancy and function of the NMDAR using a shared mechanism with α-amino-3-hydroxy-5-methyl-4-isoxazolepropionic acid receptors (AMPARs) and kainate receptors (Penn et al., 2008; Coleman et al., 2009, 2010; Scholefield et al., 2019). Although this hypothesis is supported by a limited series of mutant NMDARs (Kenny et al., 2009; She et al., 2012; Skrenkova et al., 2019), experiments involving a larger series of mutant NMDARs complicate this relatively simplistic interpretation, as the EC_50_ values for agonists are often not correlated with the surface expression of mutant NMDARs (Swanger et al., 2016). In addition, the extracellular parts of the GluN subunits form multiple disulfide bridges (Laube et al., 1993; Choi et al., 2001; Lipton et al., 2002; Furukawa and Gouaux, 2003; Papadakis et al., 2004), as well as they are robustly *N*-glycosylated in the ER (Chazot et al., 1995; Everts et al., 1997; Huh and Wenthold, 1999; Kaniakova et al., 2016); however, how these modifications contribute to the individual steps necessary for ER processing of the NMDARs remains largely unknown. The relevance of this review is emphasized by the fact that there have been identified many pathogenic mutations in the GluN subunit genes, directly linked to a variety of neuropsychiatric disorders and conditions, which could alter the ER processing of the NMDARs (Hu et al., 2016; Garcia-Recio et al., 2020).

## The ATD in GluN Subunits

The ATD in mammalian GluN subunits is an extracellular domain comprised of approximately 400 amino acids residues, with 35–57% homology among the GluN2A through GluN2D and 22% homology between GluN1 and GluN3 (Hansen et al., 2010; Romero-Hernandez et al., 2016). Structurally, the ATDs of GluN subunits are clamshell-like bi-lobed structures with two modular halves, called R1 (distal to the membrane region) and R2 (proximal to the membrane region), linked to the LBD *via* ATD-LBD linkers (Karakas et al., 2009). Importantly, ATDs have been shown to regulate the functional properties of NMDARs in a subunit-dependent manner (Yuan et al., 2009; Mesic et al., 2016). In addition, ATDs contain modulatory binding sites for endogenous ions, such as Zn^2+^ and H^+^, at both GluN1/GluN2A and GluN1/GluN2B receptors (Paoletti et al., 1997; Rachline et al., 2005; Gielen et al., 2009; Jalali-Yazdi et al., 2018). More detailed information on the structural roles that ATDs play in regulating the functional and pharmacological properties of NMDARs can be found in previously published excellent reviews (Hansen et al., 2010, 2018; Regan et al., 2015; Stroebel and Paoletti, 2020).

The ATD likely mediates the assembly of the initial GluN subunit dimers in the ER. For example, removing the first 380 residues in the GluN1 abolished GluN1-GluN1 homodimerization as well as the subunit’s association with GluN2A, leading to reduced surface delivery of GluN1/GluN2 receptors (Meddows et al., 2001). More recently, Farina and colleagues found that the Y109C and T110A mutations in the GluN1 promote homodimerization and heterodimerization, respectively (Farina et al., 2011). In addition, work by our group and others identified an ER retention signal in the A2 segment of the ATD in the GluN2A and GluN2C, but not the GluN2B; this signal is masked by the ATD in GluN1 during the formation of GluN1/GluN2 receptors (Horak et al., 2008; Qiu et al., 2009; Lichnerova et al., 2014). Although it is currently unknown whether the ATD in GluN3 contains an ER retention signal, a GluN3A lacking the ATD has reduced surface delivery of NMDARs (Skrenkova et al., 2019). With respect to pathogenic mutations, the ATDs in GluN subunits have reduced negative selection compared to the LBDs, resulting in a wide range of missense mutations in the GluN2; however, the precise effect of these mutations on the early processing of NMDARs is poorly understood (Swanger et al., 2016). We would like to emphasize the fact that it is currently unclear whether conformational changes in ATDs, including those induced by interactions with Zn^2+^ and H^+^, are sensed by ER quality control mechanisms.

## The Glycine-Binding Site in GluN1 and GluN3 Subunits

Johnson and Ascher (1987) found that glycine is a co-agonist of NMDARs. Kuryatov et al. (1994) then identified the key amino acid residues of the GluN1 for its interaction with glycine. Binding assays with isolated LBDs of the GluN1 and GluN3 showed that glycine’s affinity for the GluN1 is 26.4 μM (Furukawa and Gouaux, 2003) while it is approximately 650-fold higher for the GluN3A (Yao and Mayer, 2006), even though the LBDs in the GluN1 and GluN3 share 34% amino acid identity (Yao et al., 2008). Subsequently, structural studies showed that in the case of the GluN1, the α-carboxyl group in the glycine molecule interacts with the subunit *via* the guanidium group at R523, the amide groups at T518 and S688, and the hydroxyl group at S688, while the amino group in glycine interacts with the P516, T518, and D732 residues. In addition, the Q405 residue creates internal bonds with W731 and D732 (Furukawa and Gouaux, 2003), and the side chain of F484 residue in GluN1 forms key hydrophobic interactions with W731 and caps the binding site as a lid, thus sterically prevents the bound agonist from leaving the closed cleft conformation of the LBD (Kalbaugh et al., 2004; Inanobe et al., 2005). Similarly, the carboxyl group in the glycine molecule interacts with R638, S633, and S801 residues in GluN3A, and the amino group in glycine binds to S631, S633, and D845 residues. The GluN3A’s ligand-binding site is closed by an interaction between the E522 residue and the M844 and D845 residues and is capped by the side chain in the Y605 residue (Yao et al., 2008). Interestingly, D-serine binds the GluN1 with an affinity of ∼7 μM (Furukawa and Gouaux, 2003), and GluN3 binds D-serine with even higher affinity (Yao and Mayer, 2006). In addition, four water molecules form interactions between the glycine molecule and the LBD in the GluN1 (Furukawa and Gouaux, 2003); in the case of GluN3, three water molecules interact with the LBD (Yao et al., 2008). The mechanism of D-serine binding to GluN1 and GluN3A is similar to the mechanism for glycine binding, except that the carboxyl group in the D-serine molecule forms bonds with GluN1 and GluN3A *via* the hydroxyl groups in T518 and S633, respectively, the hydroxyl groups in S688 and S801, respectively, and the carboxyl groups in D732 and D845, respectively (Furukawa and Gouaux, 2003; Yao et al., 2008).

Consistent with the hypothesis that quality control mechanisms in the ER sense receptor’s ligand occupancy, the surface expression of NMDARs carrying mutations in the glycine-binding site in GluN1 and GluN3 were examined. Specifically, the D732A mutation in the GluN1 —which decreases the GluN1/GluN2B receptor’s affinity for glycine approximately 30,000-fold due to the disruption of the hydrogen bond between the carboxyl group of D732 and the amino group of glycine (Williams et al., 1996)—reduces the surface delivery of GluN1/GluN2A receptors by approximately 90% (Kenny et al., 2009). In addition, three other mutations in GluN1 were studied: A714L, which destabilizes the glycine-bound closed cleft conformation of the LBD of GluN1 (Furukawa and Gouaux, 2003); F484A, which lacks an aromatic ring responsible for forming hydrogen bonds in its side chain; and T518L, which disrupts the hydrogen bonds that coordinate glycine within its binding site (Yao and Mayer, 2006). In addition, GluN1 with the F484A and T518L double mutations was studied, previously found to be insensitive to glycine at concentrations up to 30 mM (Kvist et al., 2013). The mutated GluN1/GluN3A receptors exhibited reduced surface expression in contrast to wild-type receptor in the following descending order: GluN1-A714L/GluN3A, GluN1-F484A/GluN3A, GluN1-T518L/GluN3A, GluN1-D732A/GluN3A, and GluN1-F484A + T518L/GluN3A (Skrenkova et al., 2019); which is correlated with respect to the glycine EC_50_ values for GluN1/GluN2 receptors (Kvist et al., 2013) and the time constant of desensitization for GluN1/GluN3A receptors (Skrenkova et al., 2019). Similarly, the GluN1/GluN3A receptors with analogous mutations in the GluN3A were expressed at the cell surface in the following order (from highest to lowest expression): wild-type GluN1/GluN3A, followed GluN1/GluN3A-T825L, GluN1/GluN3A-Y605A, GluN1/GluN3A-S633L, GluN1/GluN3A-D845A, and GluN1/GluN3A-Y605A-S633L (Skrenkova et al., 2019; Figures 1B–D). Moreover, GluN1/GluN3A receptors in which the GluN3A contains the pathogenic D845N mutation (classified by the UCSC browser as “clinically associated”) failed to reach the cell surface and produce functional NMDARs (Skrenkova et al., 2019). The fact that all of the mutated amino acid residues in the LBD—with the exception of F484 and Y605 in the GluN1 and GluN3A, respectively—directly interact with glycine indicates that the LBD’s sensitivity for glycine is likely the sole factor that regulates the surface delivery of GluN3A-containing NMDARs. This conclusion is supported by the pathogenic S688Y mutation in GluN1—sterically preventing the binding of both glycine and D-serine to the LBD—which profoundly reduces the surface expression of GluN3A-containing NMDARs (Skrenkova et al., 2020).

## The Glutamate-Binding Site in GluN2 Subunits

The first indication of the existence of NMDARs dates back to 1963, when Curtis and Watkins tested large series of synthetic substances and one of the compounds tested was NMDA (Curtis and Watkins, 1963), which later provided the name for this group of glutamate receptors. Interestingly, as nicely reviewed previously, the effect of NMDA was known before the confirmation of L-glutamate as one of the major neurotransmitters in the mammalian CNS (Watkins, 2000). Laube et al. (1997) mutated amino acid residues in GluN2B according to sequence homology to GluN1 and the evolutionary ancestor from the bacterial leucine-arginine-ornithine binding protein (LAOBP) and they discovered the following amino acid residues involved in direct interaction with L-glutamate (which was later confirmed by crystallography): E413, H486, S512, R519, V686, and S690. Subsequently, it was shown that the LBDs of different GluN2 subunits show slightly different EC_50_ values for L-glutamate (GluN2D: ∼0.5 μM; GluN2C: ∼1.7 μM; GluN2B: ∼2.9 μM; GluN2A: ∼3.3 μM) (Erreger et al., 2007). Because only eight of the 39 amino acid residues directly lining in the ligand binding pocket are different among the GluN2 subunits (Kinarsky et al., 2005), it is unlikely that single amino acid residue replacement could be responsible for the different sensitivity of GluN2 subunits to L-glutamate (Anson et al., 1998; Chen et al., 2005; Hansen et al., 2005; Kinarsky et al., 2005; Erreger et al., 2007). The first crystal structure with the LBD of GluN2A helped to fully understand interaction between L-glutamate and NMDAR (Furukawa et al., 2005). Specifically, this study revealed that the α-carboxyl group in the L-glutamate molecule interacts with the guanidium group in the R518 residue and with the backbone amines in the S689 and T513 residues. Moreover, the amino group in L-glutamate interacts with the hydroxyl groups in the S511 and T513 residues and *via* a water molecule (W1) with the γ-carboxyl group in E413 and the hydroxyl group in Y761; in addition, the hydroxyl group in Y730 and the γ-carboxyl group in E413 forms an interaction between S1 and S2 segments (Laube et al., 2004; Maier et al., 2007). The γ-carboxyl group in the L-glutamate molecule interacts with the backbone amines in the S689 and T690 residues and with the hydroxyl group in T690, as well as *via* a water molecule (W2) with the backbone amines in E691 and G688 and with the carbonyl group in V685 (Jespersen et al., 2014). In addition, the L-glutamate molecule is stabilized in the LBD by hydrophobic interactions with the side chains in the H485 and Y730 residues (Furukawa et al., 2005), and Jespersen and colleagues predicted additional interactions between a third water molecule (W3) and the carbonyl group in V685 and between the γ-carboxyl group in the L-glutamate molecule and the D731 residue (Jespersen et al., 2014). Interestingly, the presence of the D731 residue (together with the displacement of the water molecule) leads to the selective binding of NMDA to GluN2A, in contrast to AMPA and kainate receptors, which have glutamate with a longer side chain in this homologous position (Armstrong and Gouaux, 2000; Furukawa et al., 2005; Jespersen et al., 2014).

To verify the hypothesis that the ER quality control machinery senses the correct occupation of the NMDAR by L-glutamate, She et al. prepared a series of mutated GluN2B from a previous electrophysiological study that identified amino acid residues critical for interaction with L-glutamate (Laube et al., 1997). In particular, the authors used the following mutations in GluN2B: E413A, which likely disrupts the interaction of water with the γ-carboxyl group of glutamate, and which also likely disrupts the interaction between S1 and S2 segments; F416S, which most likely alters the potency of glutamate indirectly because this amino acid is not directly involved in the interaction with L-glutamate; V686A, which has not been structurally characterized but could theoretically disrupt the interaction of GluN2B with the water molecule; S690G, which likely affects the interaction of GluN2B with the α-carboxyl group of L-glutamate due to the smaller uncharged side chain (Furukawa et al., 2005). Their elegant experiments revealed that an increase in the EC_50_ values for L-glutamate (Laube et al., 1997) negatively correlates with co-localization with GA as well as surface delivery of GluN1/GluN2B receptors, with the following order (from highest to lowest expression): wild-type GluN1/GluN2B, followed by GluN1/GluN2B-V686A, GluN1/GluN2B-F416S, GluN1/GluN2B-S690G, and GluN1/GluN2B-E413A (She et al., 2012). Interestingly, the fact that not all of these residues are involved in the interaction between the GluN2B and the L-glutamate molecule (Laube et al., 1997; Furukawa et al., 2005) supports the notion that any structural changes that affect the subunit’s sensitivity for L-glutamate reduce the surface delivery of the NMDARs. Consistent with this notion, Swanger and colleagues reported that the surface delivery of NMDARs carrying pathogenic mutations in the GluN2A LBD had the following rank order (from highest to lowest expression): wild-type GluN1/GluN2A, followed by GluN1/GluN2A-V734L, GluN1/GluN2A-I694T, GluN1/GluN2A-M705V, GluN1/GluN2A-A727T, GluN1/GluN2A-G483R, GluN1/GluN2A-V685G, and GluN1/GluN2A-D731N; the same rank order was observed with respect to the receptor’s affinity for L-glutamate (Swanger et al., 2016). With the exception of the D731N and V685G mutations, none of the above-mentioned pathogenic mutations in GluN2A likely affect direct interaction with L-glutamate. The observed effect of the V685G mutation could be explained similarly to the effect of the V686A mutation in GluN2B (above). In the case of the D731N mutation, it is likely that the altered side chain charge is a major cause of decreased NMDAR surface expression. Moreover, the pathogenic E413G mutation in GluN2B reduced the surface delivery of NMDARs by approximately 80% and increased the receptor’s EC_50_ for L-glutamate approximately 50-fold (Swanger et al., 2016), likely by promoting the unbinding of L-glutamate and opening of the LBD (Wells et al., 2018). On the other hand, other pathogenic mutations within the LBD of GluN2A (A716T, K772E, V452M, R504W, V506A, K669N, P699S, and E714K) and the LBD of GluN2B (R540H, R682C, and R696H) revealed no clear correlation between the receptor’s EC_50_ for L-glutamate and surface expression, underscoring the notion that the potency of L-glutamate is only one factor that regulates the surface delivery of NMDARs (Figures 1E–G). We would like to emphasize that existing studies have not systematically investigated homologous mutations in amino acid residues in the LBDs of GluN2A and GluN2B, so it is currently unclear whether there are differences in ER processing of GluN1/GluN2A and GluN1/GluN2B receptors.

## Disulfide Bridges in GluN Subunits

Most membrane proteins contain one or more disulfide bridges, which are important for creating the correct protein conformations (Oka and Bulleid, 2013; Okumura et al., 2015). The establishment of disulfide bridges is catalyzed in the ER, which has a suitable oxidizing environment, as well as a robust enzymatic apparatus composed of dozens of different enzymes, such as protein disulfide isomerase (PDI) and ER oxidoreductin 1 (Ero1) (Bulleid and Ellgaard, 2011; Sato and Inaba, 2012). As previously reviewed in detail, this enzymatic apparatus is a key part of the ER quality control machinery (Feige and Hendershot, 2011; Oka and Bulleid, 2013; Ali Khan and Mutus, 2014). Interestingly, the functions of key enzymes regulating production of disulfide bridges may change during neuropathological conditions, suggesting that proper formation of disulfide bridges is essential for the normal functioning of the human CNS (Andreu et al., 2012; Mossuto, 2013; Perri et al., 2015).

**FIGURE 2:**
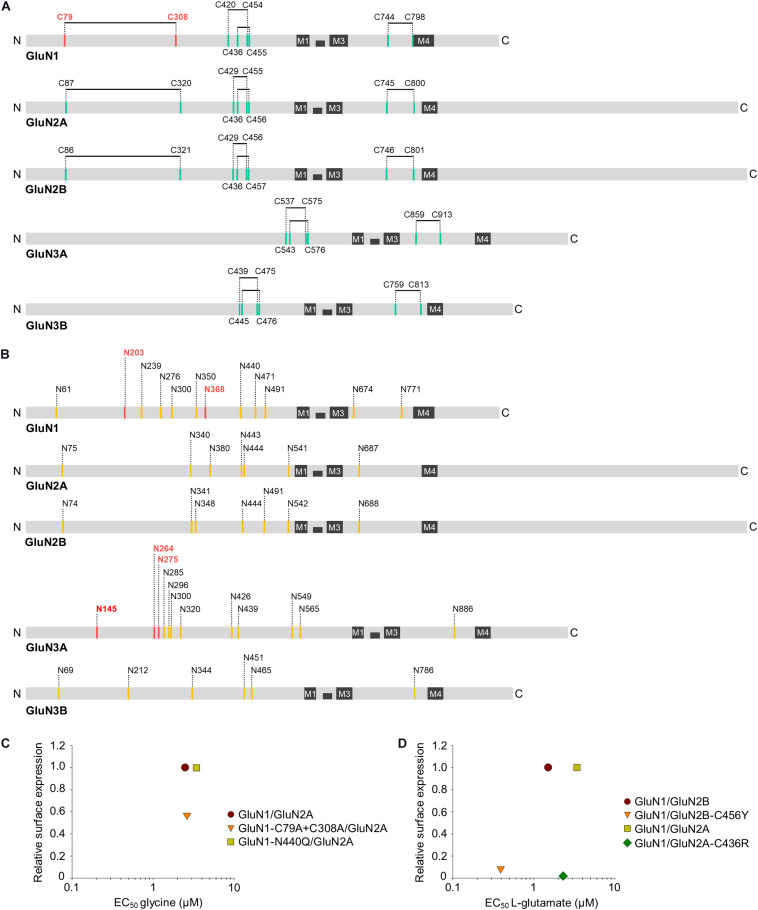
Schematic diagram showing the approximate locations of the predicted disulfide bridges **(A)** and *N*-glycosylation consensus sites [N-X-S/T; **(B)**] in the various GluN subunits. The four membrane domains (M1 through M4) are indicated, and the disulfide bridges and *N*-glycosylation sites implicated in the ER processing of NMDARs are shown in red (see text for details). **(C,D)** The relationship of surface expression of mutated GluN1/GluN2 receptors with disrupted disulfide bridges or *N*-glycosylation site with EC_50_ values for glycine **(C)** or L-glutamate **(D)** (Choi et al., 2001; Papadakis et al., 2004; Lichnerova et al., 2015; Swanger et al., 2016; Sinitskiy et al., 2017). If necessary, the values of the relative surface expression of NMDARs were obtained by calculating values from graphs from the publications using ImageJ 1.52N software (National Institutes of Health, Bethesda, MD, United States) (Schneider et al., 2012).

Previous studies have shown that the GluN1 forms disulfide bridges between the following four pairs of residues: C79-C308, C420-C454, C436-C455, and C744-C798 (Laube et al., 1993; Lipton et al., 2002; Furukawa and Gouaux, 2003; Papadakis et al., 2004); moreover, based on its sequence homology with GluN1 and structural/functional studies, the GluN2A is predicted to form the following four disulfide bridges: C87-C320, C429-C455, C436-C456, and C745-C800, and the GluN2B is predicted to form disulfide bridges between the C86-C321, C429-C456, C436-C457, and C746-C801 residue pairs (Karakas et al., 2009; Zhang et al., 2013; Karakas and Furukawa, 2014; Figure 2A). Finally, three disulfide bridges are predicted to form in the GluN3A (C537-C575, C543-C576, and C859-C913) and GluN3B (C439-C475, C445-C476, and C759-C813) (Yao et al., 2008; Grand et al., 2018). These disulfide bridges are functionally relevant, as mutating the C79A and/or C308 residue in GluN1 reduces the surface expression of GluN1/GluN2B receptors in HEK293 cells by approximately 50% (Papadakis et al., 2004); moreover, mutating the C79 and C308 residues in GluN1 increases the EC_50_ for NMDA by 25% without affecting the EC_50_ for glycine in GluN1/GluN2A receptors (Choi et al., 2001). On the other hand, mutating the C87 and C320 residues in the GluN2A has no effect on the surface delivery of GluN1/GluN2A receptors expressed in HEK293 cells or hippocampal neurons, even though these residues play a role in the homodimerization of GluN2A (Zhang et al., 2013). Interestingly, the pathogenic C436R mutation in the GluN2A and GluN2B decreased the surface expression of NMDARs by approximately 90% (Serraz et al., 2016; Swanger et al., 2016; Addis et al., 2017), although GluN1/GluN2A-C436R receptors have only a slight change in the EC_50_ for L-glutamate and glycine (Swanger et al., 2016). Similarly, the pathogenic C456Y mutation in GluN2B also decreased the surface expression of NMDARs by approximately 90%, but only slightly alters the EC_50_ for both NMDA and glycine (Swanger et al., 2016; Figures 2C,D). Thus, the presence of specific disulfide bridges and/or the receptor conformation(s) that they help stabilize—rather than a change in agonist binding due to the loss of these bridges—is likely sensed by quality control machinery in the ER. Nevertheless, this hypothesis should be tested directly in future studies, as previous studies focused primarily on the functional and pharmacological effects of disrupting disulfide bridges on GluN1/GluN2 and GluN1/GluN3 receptors at the cell surface (Laube et al., 1993; Sullivan et al., 1994; Choi et al., 2001; Grand et al., 2018).

## *N*-Glycosylation of GluN Subunits

Proteins found in the mammalian CNS contain a very high number of tissue-specific *N*-glycosylated sites, indicating the importance of *N*-glycans for the functioning of the CNS (Zielinska et al., 2010). In general, *N*-glycosylation of the nascent polypeptide is initiated in the lumen of the ER by the addition of a dolichol-linked precursor oligosaccharide and its subsequent modification to the high-mannose form of *N*-glycans; this step is important for the proper assembly and processing of proteins in the ER (Vagin et al., 2009; Moremen et al., 2012; Xu and Ng, 2015). *N*-glycans are then remodeled during their journey from ER to GA into hybrid and complex forms, by coordinated activity of several hundred specific enzymes of the glycosylation apparatus (Vagin et al., 2009). Interestingly, several thousand different glycan structures have been identified in the mammalian CNS that are likely to regulate the intracellular sorting of glycoproteins as well as interactions between cells and their external environment (Freeze, 2006; Vagin et al., 2009; Moremen et al., 2012).

The GluN1, GluN2A, GluN2B, GluN3A, and GluN3B contain 12, 7, 7, 12, and 6 predicted *N*-glycosylation sites, respectively (Everts et al., 1997; Lichnerova et al., 2015; Skrenkova et al., 2018; Hemelikova et al., 2019; Figure 2B). When expressed in HEK293 cells, 11 of the 12 predicted *N*-glycosylation sites in the GluN1 and all seven sites in the GluN2B are occupied by *N*-glycans (Kaniakova et al., 2016), and early experiments showed that *N*-glycosylation is required for the efficient expression of GluN1/GluN2A receptors in HEK293 cells (Chazot et al., 1995). We previously showed that mutating either N203 or N368 in the GluN1 reduced the surface expression of GluN1/GluN2A and GluN1/GluN3A receptors in HEK293 cells by approximately 70%; moreover, mutating both *N*-glycosylation sites reduced the surface expression of NMDARs in hippocampal neurons by approximately 80%, likely by disrupting ER processing (Lichnerova et al., 2015; Skrenkova et al., 2018). On the other hand, no individual *N*-glycosylation sites in the GluN2A, GluN2B, or GluN3A are essential for the surface delivery of NMDARs, although simultaneously mutating three specific *N*-glycosylation sites in the GluN3A reduced the surface expression of NMDARs in hippocampal neurons by approximately 40% (Lichnerova et al., 2015; Skrenkova et al., 2018). Interestingly, the simulation predicted that intra-domain interactions involving a glycan bound to the GluN1-N440 residue stabilize the closed-clamshell conformation of the LBD of GluN1, consistently with the fact that GluN1-N440Q/GluN2A receptors (which cannot be glycosylated at the GluN1-N440 position) have an EC_50_ value for glycine increased by approximately 50% (Sinitskiy et al., 2017). However, none of the studied NMDAR subtypes containing the GluN1-N440Q mutation, GluN1-N440Q/GluN2A and GluN1-N440Q/GluN3A, showed altered surface expression compared to the respective wild-type receptors (Lichnerova et al., 2015; Skrenkova et al., 2018; Figure 2C). In addition, treating cerebellar granule cells with tunicamycin, a specific inhibitor of *N*-glycosylation in the ER, reduced the surface delivery of NMDARs but had only a slight effect on their functional properties (Lichnerova et al., 2015). Finally, experiments in which hippocampal neurons were treated chronically with specific inhibitors of the *N*-glycosylation pathway revealed that *N*-glycan remodeling in the ER and GA is not required for the surface delivery of NMDARs (Hanus et al., 2016; Skrenkova et al., 2018). Thus, the mere presence of specific *N*-glycans on GluN subunits is likely assessed during the ER processing of NMDARs. We would like to point out that there is a lack of studies examining the presence of *N*-glycans on unconventional motifs and/or the presence of other glycan structures, such as *O*-glycans, during ER processing of the NMDARs.

## Concluding Remarks

In this review, we have focused on summarizing previous studies on the roles that the extracellular domains of GluN subunits play in the processing of NMDARs in the ER, including studies that tested the main hypothesis that the ER quality control machinery senses the proper ligand occupancy of the NMDARs (Kenny et al., 2009; She et al., 2012; Skrenkova et al., 2019). There are several known endogenous ligands of LBDs in GluN2 (e.g., L-glutamate, D/L-aspartate, homocysteate, and cysteinesulfinate) as well as GluN1 and GluN3 (e.g., glycine, D/L-serine, and D/L-alanine) with high affinity for NMDARs (Erreger et al., 2007; Chen et al., 2008; Dravid et al., 2010; Traynelis et al., 2010). Although the exact concentrations of these ligands in the ER are not currently known, they are likely present at sufficiently high concentrations to fully occupy newly formed wild-type NMDARs, as they play key roles in intracellular signaling and metabolic pathways (Berger et al., 1977; Fleck, 2006; Mothet et al., 2015; Cooper and Jeitner, 2016). In principle, a neuron could regulate the availability of NMDAR ligands to alter ER processing of NMDARs, but it is more likely that the ER quality control machinery verifies the proper functioning of the NMDARs fully saturated with ligands.

As we have noted, there are likely several independent mechanisms that are necessary for the proper processing of NMDARs in the ER. It remains to be clarified whether these mechanisms are used for individual steps in ER processing of NMDARs, as redundant to protect against premature release of NMDARs from the ER, or whether they are used in specific situations, such as synaptic activity. In either case, NMDARs likely interact with dozens of other proteins during their processing in the ER besides previously published ones, such as Sec8 (Sans et al., 2003), SAP102 (Standley and Baudry, 2000), and SAP97 (Jeyifous et al., 2009). It is important to emphasize that the mechanisms underlying ER processing of NMDARs have usually been investigated using mammalian cell lines, such as HEK293 and COS-1/-7 cells, and also using primary cultured mammalian neurons (Standley et al., 2000; Horak et al., 2008; Kenny et al., 2009; Qiu et al., 2009; She et al., 2012; Swanger et al., 2016; Skrenkova et al., 2019, 2020). Since the experimental results are often similar between the mentioned cell types, although the protein compositions of their ER are likely different (Ramirez and Couve, 2011; Karagas and Venkatachalam, 2019), we expect that NMDARs use predominantly general ER quality control mechanisms shared by all mammalian cells. In addition, the ER quality control machinery includes a set of specific enzymes that catalyze the formation of disulfide bridges (Oka and Bulleid, 2013; Okumura et al., 2015) as well as the *N*-glycosylation (Vagin et al., 2009; Moremen et al., 2012), but it is currently unknown which enzymatic cascades catalyze these modifications of NMDARs. Therefore, it is now necessary to focus on conducting mechanistic studies to understand how the ER quality control machinery processes NMDARs under normal physiological conditions. The fact that several pathogenic mutations in the GluN subunits alter the surface delivery of NMDARs (Swanger et al., 2016; Addis et al., 2017; Chen et al., 2017; Liu et al., 2017; Ogden et al., 2017; Vyklicky et al., 2018; Skrenkova et al., 2020), further emphasizes the importance of understanding the molecular mechanisms regulating ER processing of NMDARs also under pathophysiological conditions. Given that a wide range of ER quality control (Munshi and Dahl, 2016; Zhou et al., 2018) and NMDAR (Traynelis et al., 2010; Strong et al., 2014) modulators are currently available, it is possible that one of the potential clinical treatments of patients with abnormal regulation of NMDARs could be pharmacologically induced alteration of the ER processing of the NMDARs.

## Author Contributions

MH, PB, JN, EL, PR-R, and KR wrote the manuscript. JN and KR made the figures. All authors contributed to the article and approved the submitted version.

## Conflict of Interest

The authors declare that the research was conducted in the absence of any commercial or financial relationships that could be construed as a potential conflict of interest.

## Abbreviations

AMPA: 2-amino-3-(5-methyl-3-oxo-1,2-oxazol-4-yl) propanoic acid
ATD: amino-terminal domain
CNS: central nervous system
COPII: coat protein complex II
COS: the cells being CV-1 in Origin and carrying the SV40 genetic material
CTD: C-terminal domain
ER: endoplasmic reticulum
Ero1: ER oxidoreductin 1
GA: Golgi apparatus
HEK293: human embryonic kidney 293 cells
LAOBP: leucine–arginine–ornithine binding protein
LBD: ligand-binding domain
NMDAR: *N*-methyl -D-aspartate receptor
NTD: N-terminal domain
PDB: Protein Data Bank
PDI: protein disulfide isomerase
PSD-MAGUKs: postsynaptic density (*PSD*)-95 family of membrane-associated guanylate kinases.
